# Clinical‐dosimetric relationship between lacrimal gland dose and keratoconjunctivitis sicca in dogs with sinonasal tumors treated with radiation therapy

**DOI:** 10.1111/jvim.15744

**Published:** 2020-02-22

**Authors:** Valerie J. Poirier, Arata Matsuyama, Changseok Kim, Johnson Darko, Andre Fleck

**Affiliations:** ^1^ School of Veterinary Science Massey University Palmerston North New Zealand; ^2^ Animal Cancer Centre Ontario Veterinary College, University of Guelph Guelph Ontario Canada; ^3^ Department of Biomedical Sciences Ontario Veterinary College, University of Guelph Guelph Ontario Canada; ^4^ Department of Medical Physics Grand River Regional Cancer Centre Kitchener Ontario Canada

**Keywords:** dry eye syndrome, IGRT, IMRT, nasal tumor, organ at risk, xerophtalmia

## Abstract

**Background:**

Dogs with sinonasal tumor can develop keratoconjunctivitis sicca (KCS) after radiation therapy (RT). In humans, the incidence of xerophtalmia is associated with the mean radiation dose received by the ipsilateral lacrimal gland (LG).

**Hypothesis/Objectives:**

The eyes receiving a higher mean LG dose are more likely to develop KCS. The aim of the study was to determine a starting threshold dose to use as dose constraint for intensity‐modulated radiation therapy (IMRT).

**Animals:**

Dogs with nasal tumors treated with RT between August 2013 and December 2016.

**Methods:**

Case control retrospective study of dogs with sinonasal tumor treated with 42 Gray (Gy) in 10 fractions using IMRT. Dogs were included if development of KCS after RT was documented (cases) or adequate follow‐up information with Schirmer tear test (STT) result for ≥6 months after RT was available (controls). Lacrimal glands were contoured and dose distribution was calculated using the original treatment plan to determine prescribed doses to LGs.

**Results:**

Twenty‐five dogs were treated with RT and 5 dogs (20%) developed KCS. Fifteen dogs met the inclusion criteria including 5 unilateral KCS and 10 control dogs, resulting in 5 KCS eyes and 25 control eyes. KCS developed at a median of 111 days (84‐122) after 1st RT. The mean LG dose reached using a 4.2 Gy per fraction was 33.08 Gy (range: 23.75‐42.33) for KCS eyes and 10.33 Gy (1.8‐24.77) for control eyes (*P* < .001). The minimum LG mean dose for developing KCS was 23.75 Gy. No eyes that received a mean LG dose <20 Gy developed KCS versus 5/7 (71%) developed with >20 Gy.

**Conclusion and Clinical Importance:**

Contouring and applying a dose constraint on LGs should be performed when using IMRT in dogs with sinonasal tumors to reduce the risk of KCS.

**Abbreviations**
2D: 2 dimensional
BED: biological effective dose
CBCT: cone beam computerized tomography
CT: computerized tomography
CTV: clinical target volume
EQD2: equivalent dose in 2 Gy fractions
GTV: gross tumor volume
Gy: gray
IGRT: image guided radiation therapy
IMRT: intensity‐modulated radiation therapy
KCS: keratoconjunctivitis sicca
LG: lacrimal gland
OAR: organ at risk
OD: right eye
OS: left eye
PTV: planned target volume
RT: radiation therapy
STT: Schirmer tear test
TEG: accessory lacrimal gland of the third eyelid
VRTOG: Veterinary Radiation Therapy Oncology Group

## 1 INTRODUCTION

Radiation therapy (RT) is accepted as the standard of care for local control of sinonasal tumors in dogs.^1^ Use of intensity‐modulated radiation therapy (IMRT) allows radiation to be delivered with greater conformity as well as to reduce the dose to the organs at risk (OAR).^2^, ^3^, ^4^ The OARs are nontarget tissues that could suffer important morbidity if exposed to radiation. In human's threshold or volume‐related dose constraints such as Quantitative Analyses of Normal Tissue Effects in the Clinic (QUANTEC)^5^ exist that predict the level of damage to certain tissues. These thresholds are less well established in veterinary medicine. Xerophtalmia (dry eye syndrome/Keratoconjunctivitis sicca (KCS)) is a condition characterized by insufficient tear production and can be secondary to lacrimal gland (LG) atrophy; it is a well‐described adverse event of RT^6^, ^7^, ^8^, ^9^ The incidence of KCS in the original proof of principle study on ocular sparing with the use of IMRT for the treatment of dogs with sinonasal tumor was 30% when using a 2D planning system and Cobalt^60^ versus 3% with IMRT and TomoTherapy.^3^ Treatment of dogs with sinonasal tumor with IMRT using a linear accelerator has a 42% incidence of KCS in the ipsilateral eye.^2^

In human radiation oncology, a dose‐response relationship between maximum dose to the LG and the development of KCS is established and that is an increase in KCS incidence was demonstrated as the LG mean dose rises to greater than 15 to 24.9 Gray (Gy). 10 There is a sigmoid dose‐response curve showing a 0% incidence of KCS if dose to the lacrimal gland was <30.0 Gy and a 100% incidence if >57.0 Gy in 2 Gy/fraction protocol.^11^ However, because the difference in canine and human anatomy, the dose‐response relationship for the lacrimal gland and the development of KCS could be different.

Our hypothesis was that the eyes receiving a higher mean LG dose will be more likely to develop KCS and the aim of the study was to determine a starting threshold dose to use as constraint for IMRT planning for the LGs.

## 2 METHODS AND MATERIALS

### 2.1 Case selection

This is a retrospective case control study. The medical records of dogs with sinonasal tumors undergoing RT with 10 daily fractions of 4.2 Gy using IMRT/Image guided radiation therapy (IGRT) between August 2013 and December 2016 at the Animal Cancer Centre of the University of Guelph were reviewed. Inclusion criteria included development of KCS (diseased eyes) and/or normal Schirmer tear test (STT) (>15 mm/min), more than 6 months after irradiation (control eyes). STT used in our clinic is the STT I (Eagle vision, Memphis, Tennessee). The STT I is performed by placing a standardized 5 mm 9 35 mm strip of Whatman no. 41 filter paper in the ventral conjunctival fornix for 60 seconds, without the use of topical anesthesia.^12^ KCS is usually suspected when STT results are below 15 mm/min and are associated with ophthalmic signs, such as hyperemia, mucoid discharge accumulation, and keratitis.^12^ Exclusion criterion was preexisting ocular disease. Information retrieved from the medical records were: breed, weight, sex, tumor histology, modified Adams stage retrospectively; Stage 1: Confined to 1 nasal passage with no bone involvement beyond turbinates; Stage 2: Any bone involvement with no evidence of orbit/subcutaneous/submucosal mass; Stage 3: Orbit involved or nasopharyngeal or subcutaneous or submucosal mass; and Stage 4: Tumor‐causing lysis of the cribriform plate,^3^ gross tumor volume (GTV), clinical tumor volume (CTV), planned target volume (PTV), acute and late ocular toxicity according to the Veterinary Radiation Therapy Oncology Group radiation morbidity scoring scheme,^13^ results of STT, date of KCS diagnosis, time between first RT and development of KCS, and time between first RT and last normal STT for control group.

### 2.2 Positioning and planning CT

Dogs were routinely immobilized in a vacuum deformable mattress (Vac‐Lok, CIVCO, Orange City, Iowa) and indexed bite block (3M‐ Express STD Putty ESPE Dental Products, St. Paul, Minnesota) for the planning CT and for each of the RT session. All dogs were kept in sternal recumbency with forelimbs extended caudally and elbows extended. A 16‐slice helical GE Bright speed CT (GE Healthcare, Little Chalfont, UK) was used for the planning CTs. The kV and mA used were 120 and 160, respectively. Slice thickness was 2 mm and a soft tissue and bone algorithms were used for reconstruction. All dogs had some form of bolus placed over the nose that did cover the entire head including the eyes. The bolus could vary from a home‐made bolus to commercial bolus. The bolus was present for the planning CT and for every RT.

### 2.3 ORIGINAL RADIATION PLANNING

All plans were generated using the Eclipse Planning System Version 11 (Varian Medical Systems, Palo Alto, California) using AAA algorithm. All dogs had a structure set created. All dogs were contoured by an American College of Veterinary Radiology board certified radiation oncologist. The usual contouring technique was GTV contoured on CT after contrast in a bone window, and 1 cm CTV expansion confined to the nasal cavity but including the entire nasal bone (unless extra nasal mass was present then CTV was on a case‐by‐case basis) and the frontal sinus fluid if present. PTV expansion was a 3D‐2 mm from the front of the eyes caudally and 3D‐4 mm cranial to the eyes to account for pitch during radiation. OAR that was routinely contoured on the original plan included the right eye (oculus dexter‐OD), left eye (oculus sinister‐OS), left ocular lens, right ocular lens, and brain. The prescription for all dogs was 42 Gy in 10 fractions on a Monday to Friday schedule. The plan was normalized to ensure that 95% of PTV was receiving 95% of the prescribed dose. Effort was made to reduce the dose to eyes but target coverage was considered more important. No effort was made to limit the dose to the LGs at the time of original planning.

### 2.4 RADIATION TREATMENT

Dogs were anesthetized and positioned for their treatment by radiation therapists. On board kV cone beam computed tomography (CBCT) imaging using the ix2300 Clinac linear accelerator (Varian Medical Systems, Palo Alto, California) was used for patient alignment to verify target positioning at each radiation session. CBCT settings used the “high quality head” setting with a 2.5 mm slice thickness with an overlap of 2 mm, 512 × 512‐pixel field of view and a full‐fan bowtie filter. The volume of interest for matching during treatment was brain/eyes at the level of the PTV and the match was confirmed by at least 2 persons; either 2 human‐trained radiation therapists or a veterinary radiation oncologist and a human‐trained radiation therapist.

### 2.5 FOLLOW‐UP

The standard recommended follow‐up for dogs with sinonasal tumors is a recheck every 2 weeks after radiation until acute adverse effects have resolved then every 3 months for a physical examination and if the owner wishes a follow‐up advanced imaging. During these follow‐up visits, it was at the discretion of the attending veterinarian to perform a STT and as STT was not routinely performed on all dogs, we elected to only include dogs for our control group with a normal STT as a more objective measure then a note in the medical record stating that the eyes were normal.

### 2.6 CONTOURING OF THE LACRIMAL GLAND

On the dogs that were included in the study, a duplicate of the original structure (“lacrimal”) set was created in the Eclipse planning system and 4 extra structures were created retrospectively and contoured for each dog: left LG, right LG, right TEG (accessory lacrimal gland of the third eyelid), and left TEG. The LGs and TEGs were contoured in the axial plane after contrast CT scan in a soft tissue window.^14^ The volume of each LG and TEG was obtained.

### 2.7 LACRIMAL DOSE CALCULATION

The “lacrimal” structure set was used with the original plan parameters to generate a dose distribution for the new structure set. For each dog, it was verified that the dose distribution was identical for all the original structures. Using the dose volume histogram for each LG, dosimetric parameters evaluated were: mean dose, maximum dose, V15 (volume of LG receiving 15 Gy), V20 (volume of LG receiving 20 Gy), and V25 (volume of LG receiving 25 Gy). Mean dose to both eyes (OS and OD), both TEG as well as the PTV were recorded.

### 2.8 STATISTICAL ANALYSIS

Descriptive statistics were used for continuous data with mean or median values pending normality and categorical data with frequencies. The 2 groups were analyzed for differences in the following factors: LG V15, LG V20, LG V25, LG mean, LG max, TEG mean, TEG V20, eye mean dose, and PTV. The variables were first analyzed for normality using the Shapiro‐Wilke test. Either a 2‐sample test or Mann‐Whitney test was used for parametric data and nonparametric data, respectively. The incidence of KCS was calculated after stratification based on the mean LG dose. A *P* value <.05 was considered statistically significant. All statistical analyses were performed using a commercially available software program (SPSS Version 23.0, SPSS Inc., Chicago, Illinois).

## 3 RESULTS

A total of 25 dogs were treated during the study period. All dogs completed the radiation protocol within the 11 days prescribed. Five dogs (20%) developed KCS (all had a STT <7 mm), all of which were unilateral (OD = 3, OS = 2). Two dogs were excluded because of preexisting ocular disease (a Pug with KCS before RT and a Shih Tzu with glaucoma), and 8 dogs were excluded as a STT had not been performed during the follow‐up but none of them had reported ocular disease. Subsequently 10 dogs met the inclusion criteria for control, resulting in a total of 5 KCS eyes and 25 control eyes. A total of 12 breeds were represented; 3 Mixed breed dogs, 2 West Highland White Terrier, and 1 of each of the following: Fox Terrier, Viszla, Labrador Retriever, Husky, Airedale, Pug, Schnauzer, Brittany spaniel, and Golden Retriever. The rest of the dog characteristics are found in Table 1. The median time to KCS development was 111 days (range: 84‐122 days). The median time between the first RT and the last normal STT for the control dogs was 383 days (range: 202‐842 days). All dogs that developed KCS experienced acute ocular disease (grade 1 or 2 conjunctivitis) in the affected eye, while acute ocular toxicity (grade 1 conjunctivitis) was reported for only 1 of the control dogs that is also the longest follow‐up dog (842 days).

**Table 1:** Characteristics of dogs with sinonasal tumors

Age (years)
Median: 11 (6.5‐11)
Weight (kg)
Median: 22.2 (9‐47)
Sex
Female spayed n = 8
Male neutered n = 7
Histology
Carcinoma n = 10
Chondrosarcoma n = 4
Osteosarcoma n = 1
Stage modified Adams
1 n = 2
2 n = 5
3 n = 1
4 n = 7
Gross target volume (cm^3^)
Median: 36.8 (2.5‐104.5)
Clinical target volume (cm^3^)
Median: 132.9 (33.26‐292.3)
Planned target volume (cm^3^)
Median: 182.1 (54.4‐450.8)

The mean lacrimal volume was 0.14 cm^3^ (range: 0.07‐0.29 cm^3^) which is similar to the published normal LG data.^14^ Overall, the dose to the ipsilateral LG was significantly (33.1 ± 8.3 Gy versus 9.3 ± 5.8 Gy) larger for eyes that developed KCS versus control eyes (*P* < .001). The mean dose to the ipsilateral TEG was significantly larger (39.2 ± 4.2 Gy versus 23.1 ± 10.1 Gy, *P* < .001) for eyes that developed KCS but the ipsilateral TEG V20 was not significantly different (92% versus 58%, *P* = .06). The dose to the affected eyes as a whole and was also significantly larger (*P* < .001) whereas the PTV volume between affected dogs and control was not significantly different (Table 2). The minimum LG mean dose for developing KCS was 23.75 Gy (range: 23.75‐42.3 Gy). No eyes that received a mean lacrimal dose under 20 Gy developed KCS while 5/7 (71%) eyes receiving a mean lacrimal dose above 20 Gy developed KCS (Table 3).

**Table 2:** 

Dosimetric parameters	KCS+ eyes	KCS− eyes	*P* value
LG V15 (%)	Mean: 100	Mean: 20.6 Range: 0‐100	<.001
LG V20 (%)	Mean: 100	Mean: 8.19 Range: 0‐96	<.001
LG V25 (%)	Mean: 75.8 Range: 25.9‐100	Mean: 2.0 Range: 0‐41.1	<.001
LG Mean (Gy)	Median: 34.7 Mean: 33.1 Range: 27.75‐42.3 SD: 8.3	Median: 9.3 Mean: 10.3 Range: 1.8‐24.8 SD: 5.8	<.001
LG Max (Gy)	Median: 39.9 Mean: 36.9 Range: 27.5‐42.9 SD: 6.6	Median: 12.9 Mean: 14 Range: 3.1‐36.4 SD: 8.3	<.001
TEG Mean (Gy)	Median: 40.3 Mean: 39.2 Range: 34.0‐44.5 SD: 4.2	Median: 22.6 Mean: 23.1 Range:18.9‐27.2 SD: 10.1	.002
TEG V20 (%)	Mean: 92 Range: 60‐100	Mean: 58 Range: 0–100	0.06
Eye mean dose (Gy)	Median: 28.9 Mean: 30.8 Range: 23.8‐42.9 SD: 8.0	Median: 10.2 Mean: 11.6 Range: 5.1‐23 SD: 5.4	<.001
PTV (cm^3^)	Median: 98.4	Median: 190.7	.13

Abbreviations: cm, centimeter; Gy, gray; LG V15, lacrimal gland volume that received 15 Gy; LG V20, lacrimal gland volume that received 20 Gy; LG V25, lacrimal gland volume that received 25 Gy; LG, lacrimal gland; PTV, planned target volume.

**Table 3:** 

Mean lacrimal gland dose	Development of keratoconjunctivitis sicca
<10 Gy	0% (15/15)
10.1‐20 Gy	0% (9/9)
20.1‐30 Gy	50% (2/4)
30.1‐40 Gy	100% (2/2)
>40 Gy	100% (1/1)

## 4 DISCUSSION

The main goal of this study was to investigate the association between the ipsilateral LG radiation dose and the development of KCS after RT for the treatment of dogs with sinonasal tumors and the aim was to define a threshold dose to apply as a LG dose constraint when performing IMRT planning to decrease the likelihood of KCS development. None of the dogs that received a mean LG dose below 20 Gy developed KCS; we elected to propose this dose as a starting point knowing the limitation of this small series with only 5 dogs developing KCS and 2 dogs receiving a mean LG dose above 20 Gy but below 25 Gy that did not develop KCS. In human radiation oncology, the mean LG dose limit to avoid KCS is 15 to 24.9 Gy given as 1.8 to 2 Gy per fraction. Because of the difference in fraction size, we cannot directly compare the doses with a 4.2 Gy per fraction protocol. To compare them, we can use the equivalent dose in 2 Gy fractions (EQD2) formula: D (d + α/β/[2+ α/β]) where D is the total dose and d is the dose per fraction.^15^, ^16^ The reported α/β ratio of LG is 3.^17^ The 20 Gy in 4.2 Gy fraction using the EQD2 formula for the canine protocol would be equivalent to 28.8 Gy if given in 2 Gy fraction and be higher than the human dose limit of 15 to 24.9 Gy. The EDQ2 for the human dose limit using a 4.2 Gy per fraction would be 10.4 to 17.3 Gy. It is possible that the LG of dogs is more radio‐resistant. In our study, the mean dose to the accessory lacrimal gland of the third eyelid was also significantly higher for the KCS eyes but the V20 (volume of the TEG getting 20 Gy) was not significantly different between the KCS positive eye and the control. While the TEG might play a role in the development of KCS in these dogs, the location of the gland in the median canthus makes it much closer to the tumor and much likely to be obliterate after radiation.

The incidence (20%) of the development of KCS is our canine population is much higher than the reported incidence with an identical protocol using TomoTherapy (3%) whereas it is better than the reported incidence of 42% in dogs treated with IMRT using a linear accelerator but with a protocol of 54 to 63 Gy in 3 Gy/fraction.^2^, ^3^ It is possible that the use of IGRT using a CBCT during each radiation fraction that was used in both studies using the 42 Gy in 10 fractions protocol did lead to the significant decrease of the incidence of KCS by making possible a smaller PTV expansion and a better radiation positioning. The difference could also be explained by the difference in protocol with a total dose of 54 to 63 Gy in 3 Gy/fraction versus the 42 Gy in 4.2 Gy/fraction. To compare the protocol, the biological effective dose (BED) formula can be used; BED = D (1 + d/α/β), where D is the total dose and d is the dose per fraction. The BED for the protocols 54 to 63 Gy in 3 Gy/fraction was higher: 108 to 126 Gy versus 100.8 Gy. The difference between both studies using the same radiation protocol is difficult to determine but it could be because of a difference in calculation algorithm that may contribute to a difference in LG dose but the delivery systems are vastly different and that would probably result in a more significant difference. It is well recognized that IMRT/IGRT treatment using a TomoTherapy machine often leads to a better sparing of normal tissue.^18^, ^19^ There is also the possibility that the contours used in the TomoTherapy study were more conservative. Furthermore, the bolus used in our cases will inherently result in more superficial dose regardless of optimization and IMRT delivery.

The case control study design was selected for this study as the observation was made in our study population that the incidence of KCS was unexpectedly high when compared to a similar protocol using TomoTherapy and prompted a change in our practice where we started contouring the LGs and putting the human dose constraint during planning after December 2016.^3^ We felt that we had treated enough dogs before the change to derive significant data and that we had a good consistent follow‐up. Three major limitations of the current study are lack of complete ophthalmologic examination in all dogs, the small sample size, and the retrospective nature of the study. STT were not performed routinely before the RT. We did not have records of a normal STT for the cases that developed KCS before RT. STT is a brute quantitative measurement of tear production and does not give any indication of the tear film quality.^20^ However, the dogs that developed KCS had severe signs that made the owner perceive a decreased quality of life that were not present before radiation while none of the control dog owners or the veterinarians described ocular adverse effects on subsequent visits. The onset of KCS was fairly uniform in all dogs affected around 3 to 4 months after radiation. It appears that the LG might have a similar response to radiation as the salivary gland. Both are tissues that should react like late responding tissue because of their low α/β ratio and the functional (ie, excretory, acinar) cells of the salivary/lacrimal glands are highly differentiated and have a slow turnover, but they behave like acute responding tissues to radiation as the adverse events are seen weeks to months after RT. The exact mechanism for this response to RT for both tissues is still unknown.^17^, ^21^, ^22^ Another limitation is the possible lack of resolution/sensitivity to delineate the actual LG in some dogs although we felt we could see them in all studies and the possible limitation associated with the small structure volume for dosimetry.^23^ This is unfortunately not something that could be changed in the immediate future and is a constraint.

We conclude that contouring and applying a dose constraint on LGs should be performed when using IMRT in dogs with sinonasal tumors to prevent KCS. A dose constraint on LG of a mean dose of <20 Gy when using a 4.2 Gy per fraction protocol seems to be a good sta**r**ting point although a prospective clinical trial would need to be conducted to confirm this finding.

## CONFLICT OF INTEREST DECLARATION

Authors declare no conflict of interest.

## OFF‐LABEL ANTIMICROBIAL DECLARATION

Authors declare no off‐label use of antimicrobials.

## INSTITUTIONAL ANIMAL CARE AND USE COMMITTEE (IACUC) OR OTHER APPROVAL DECLARATION

Authors declare no IACUC or other approval was needed.

## HUMAN ETHICS APPROVAL DECLARATION

Authors declare human ethics approval was not needed for this study.
